# Study on Cutting Chip in Milling GH4169 with Indexable Disc Cutter

**DOI:** 10.3390/ma14113135

**Published:** 2021-06-07

**Authors:** Gensheng Li, Chao Xian, Hongmin Xin

**Affiliations:** 1 College of Vehicle and Traffic Engineering, Henan University of Science and Technology, Luoyang 471003, China; ligensheng@haust.edu.cn; 2 Key Laboratory of Aero-engine High Performance Manufacturing, Ministry of Industry and Information Technology, Northwestern Polytechnical University, Xi’an 710072, China; xianchao1994@163.com; 3 Hubei Key Laboratory of Power System Design and Test for Electrical Vehicle, Hubei University of Arts and Science, Xiangyang 441053, China

**Keywords:** cutting parameters, chip parameters, models

## Abstract

The study and control for chip have a significant impact on machining quality and productivity. In this paper, GH4169 was cut with an indexable disc milling cutter. The chips corresponding to each group of cutting parameters were collected, and the chip parameters (chip curl radius, chip thickness deformation coefficient, and chip width deformation coefficient) were measured. The qualitative relationship between the chip parameters and cutting parameters was studied. The quadratic polynomial models between chip parameters and cutting parameters were established and verified. The results showed that the chip parameters (chip curl radius, chip thickness deformation coefficient and chip width deformation coefficient) were negatively correlated with spindle speed; chip parameters were positively correlated with feed speed; chip parameters were positively correlated with cutting depth. The maximum deviation rate between measured values and predicted values for chip curl radius was 9.37%; the maximum deviation rate for cutting thickness deformation coefficient was 13.8%, and the maximum deviation rate of cutting width deformation coefficient was 7.86%. It can be seen that the established models are accurate. The models have guiding significance for chip control.

## 1. Introduction

In the cutting process, long and continuous chips will wind around the workpiece, tool, or fixture, which will hinder the positioning and clamping of the workpiece, reduce the quality of the machined surface, aggravate the tool wear or damage, and even affect the safety of the operator. Sometimes the machine has to stop cutting to clean the chips, which greatly reduces productivity. In addition, long and messy chips are not easy to clean and transport. However, if the chip is too small, the chip will splash and accumulate everywhere, causing cutting vibration and premature tool damage, which will also affect the processing quality and endanger the safety of the operator. Therefore, the study and control of chip is a key problem to ensure the processing quality and improve productivity in machining, especially in automatic production.

According to the formation parameters of chip, Japanese scholar Nakayama et al. [1,2] calculated the shape parameters of the spiral chip and established the chip geometry. By measuring the geometry size of the chip, the chip flow direction, up curl radius, and transverse curl radius can be calculated. Kharkevich et al. [3,4,5,6] defined six kinds of up curl radius and transverse curl radius on basis of the research of Nakayama Ichio and determined the above six kinds of up curl radius and transverse curl radius according to the geometric parameters of spiral chip, which enriched the chip shape geometry. Chen et al. [7,8] held that: in the actual cutting process, due to the constraints of tool shape and cutting space, the chips not only produce upward curl and transverse curl but also twist. They studied the situation when the chips flow out along the rake face of straight edge and flat rake face tools and described the possible shape of chips in the cutting process from a mathematical point of view. Li et al. [9,10] systematically studied the constraint relationship between the chip and the obstacle based on the spatial motion trajectory of the chip, established the constraint equation between the chip and the obstacle surface in the process of chip formation, and established the mathematical models of upward curling and transverse curling according to the chip breaking conditions.

Fang and Jawahir [11] verified the prediction formulas of chip curl radius, chip outflow angle, and cutting thickness by cutting medium carbon steel with P20 cemented carbide tool. Zhang and Peklenik [12] studied the chip flow direction and curl radius with a large number of experiments and pointed out that the ratio of the limit curvature radius of the chip to the original radius (also known as the chip radius ratio) falls in the range of 1.2 to 2.0, and the obtained chip has no effect on the cutting process and the machined surface of the workpiece. Li et al. [13] observed the chip formation process of easy cutting steel under the scanning electron microscope and achieved comprehensive research on metal chip formation mechanism, micro, quantitative, dynamic, and multiple factors.

Worthington and Redford [14,15] studied the influence of geometric parameters of chip breaking groove, edge width, on chip breaking, and gave the range of edge width, which is conducive to chip breaking, and established the model of chip curl radius. Nedes et al. [16] established a mathematical model based on chip flow angle, effective rake angle, and chip curl radius. Zhang et al. [17] held that the cutting force imposed on the shear plane of the chip and the cutting force imposed on the contact plane are not in a straight line, so there is a cutting bending moment, which is the cause of chip bending. According to the theory of slip line field, the curl radius of the chip is calculated. Ramalingam et al. [18] gave a calculation model of chip curl radius in orthogonal cutting.

Maruda et al. [19] studied the tool wear and chip shape in turning AISI 1045 steel with a sintered carbide P25 tool under three cooling conditions: dry machining, MQCL method, and MQCL + EP/AW. The results showed that MQCL + EP/AW cooling mode had the least tool wear and better chip shape. Singh et al. [20] established quadratic polynomial models of chip reduction coefficient (CRC), surface roughness, and chip tooth height with respect to cutting speed, feed speed, and cutting depth by using the response surface method and carried out multi-objective parameters optimization in turning AISI 4340 steel with the yttria-stabilized zirconia toughened alumina (Y-ZTA) ceramic cutting tool. Das et al. [21] studied the influence of cutting depth, feed speed, and spindle speed on machining forces and chip thickness when turning 4340 alloy steel with three lubrication conditions: compressed air, water-soluble coolant, and nanofluid. The quadratic polynomial models of cutting force and cutting thickness with respect to cutting depth, feed rate, and spindle speed were established. It showed that nanofluid produced the best performance in comparison to compressed air and water-soluble coolant. Iwata et al. [22] calculated the chip thickness, curl radius, and strain distribution by finite element simulation. Combining the finite element model with the ductile fracture criterion, the chip fracture was predicted. In the above literatures, the mathematical model for chip curl radius is derived from related hypotheses and theories, but many parameters in the model are difficult to obtain accurately. This paper studies the qualitative relationship between chip parameters (curl radius and deformation coefficient) and cutting parameters and establishes quadratic polynomial models between chip parameters (curl radius and deformation coefficient) and cutting parameters, which can be used for the prediction of chip parameters.

## 2. Workpiece and Milling Cutter

### 2.1. Workpiece

GH4169 is a common material for blisk manufacturing, which has good fatigue performance, high strength, and good thermal stability. The material used in this test was GH4169 (Shaanxi Changyu Aviation Equipment Co., Ltd., Xi’an, China), which is a solid solution treated after forging. The size of workpiece was 265 mm × 165 mm × 23.5 mm. Nickel base superalloy GH4169 is a superalloy with Ni as the base element, in which the content of Ni is more than 50%. Its main chemical composition is shown in Table 1 [23]. Ni has very good stability and can be stably dissolved with other metal elements, which is beneficial to increase the service life of the alloy. The chemical composition of GH4169 determines its good physical and mechanical properties, which are shown in Table 2 [23].

### 2.2. Milling Cutter

**Figure 1:**
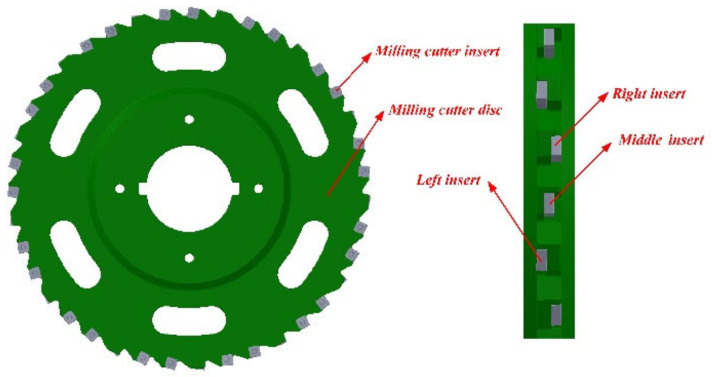
Disc milling cutter of indexable three-sided inserts.

The milling cutter used for the experiment was a disc milling cutter of indexable three-sided inserts, which consisted of a cutter disc and inserts connected by screws. There were 39 inserts, 13 left inserts, 13 right inserts, and 13 middle inserts, as shown in Figure 1. The left insert, right insert, and middle insert are staggered, which means their axes of symmetry do not coincide. The tool manufacturer is Zhuzhou Diamond Cutting Tools Co., Ltd., China. The spindle speed should not be greater than 200 r/min. When the unit of the spindle speed is r/min, and the unit of the feed speed is mm/min, the value of the feed speed must be smaller than the spindle speed.

**Figure 2:**
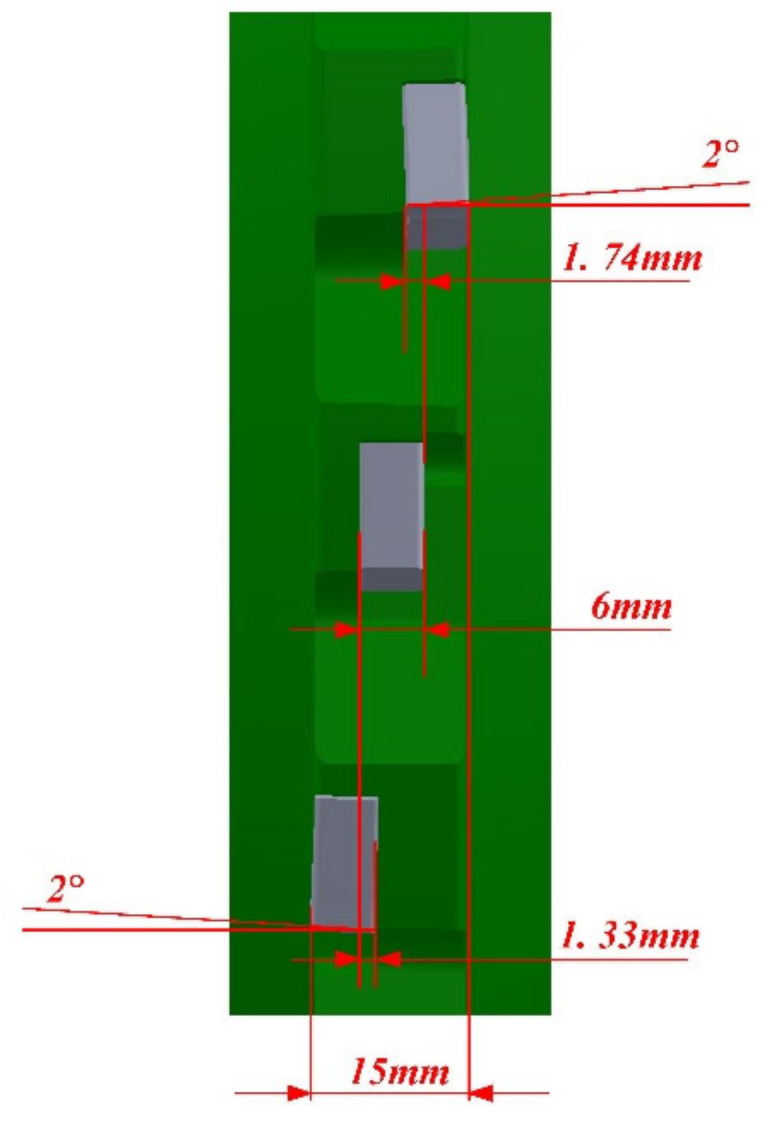
Position relationship of the three inserts.

The angle between the cutting edge of inserts and the horizontal plane was 2.2 degrees, as shown in Figure 2. So, when the milling cutter was machining the workpiece, it was an oblique cutting process. So, it was an orthogonal cutting process when the middle insert was cutting the workpiece; it was an oblique cutting process when the left insert and right insert were cutting the workpiece.

**Figure 3:**
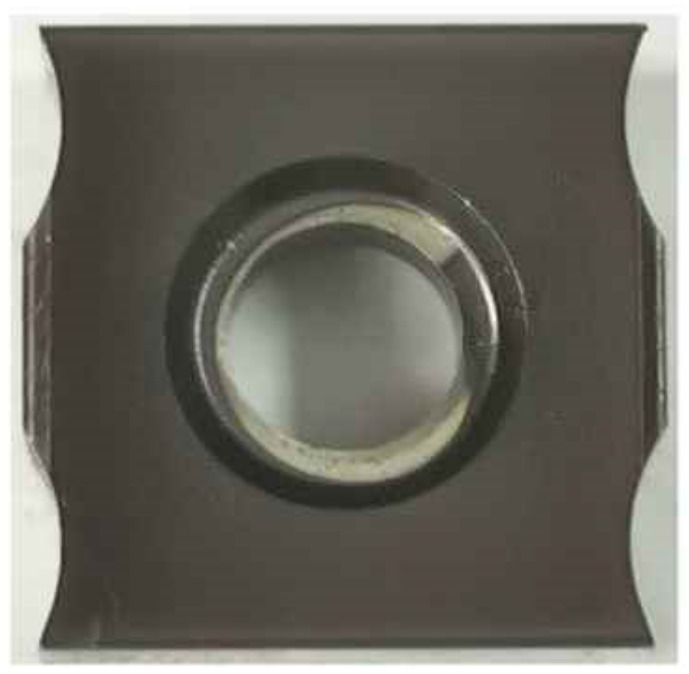
Insert.

**Figure 4:**
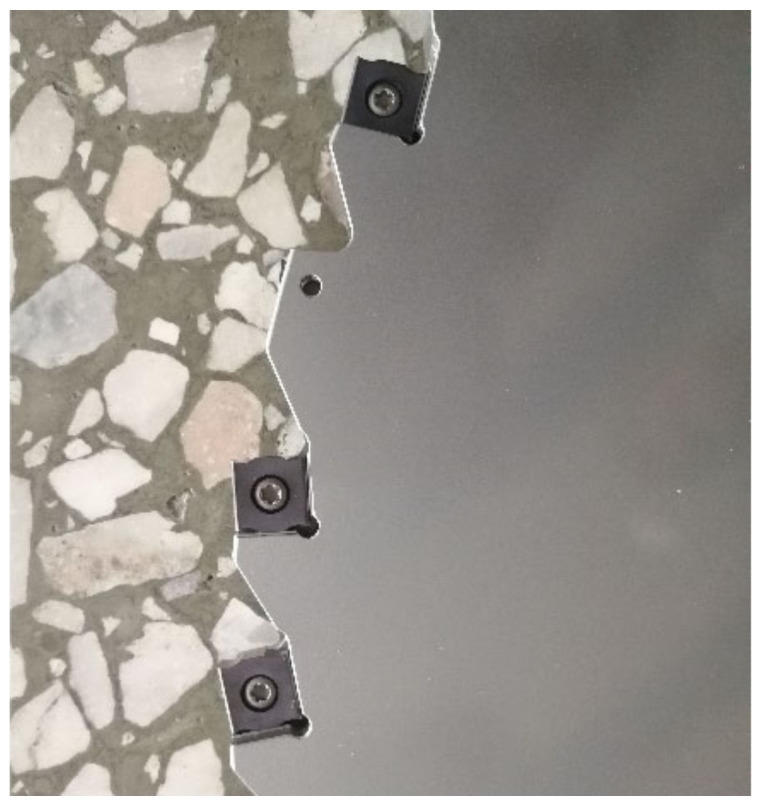
The positioning and clamping mode of inserts and cutter disc.

The insert material was carbide with a physical coating on the surface. Figure 3 displays the geometry of the insert, and the thickness was 4.3 mm. The positioning and clamping mode of inserts and cutter disc is shown in Figure 4. The milling cutter parameters are shown in Table 3.

## 3. Cutting Experiment

**Figure 5:**
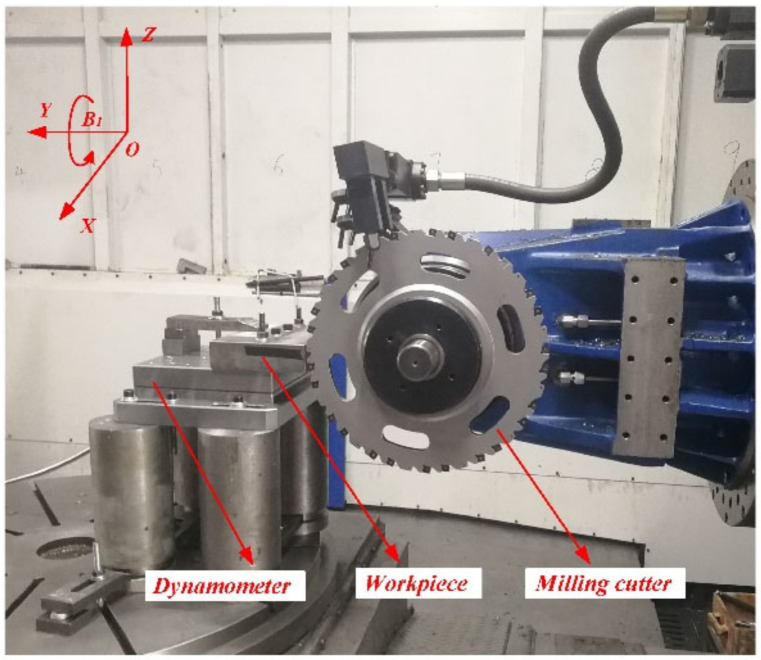
Symmetrical milling.

**Figure 6:**
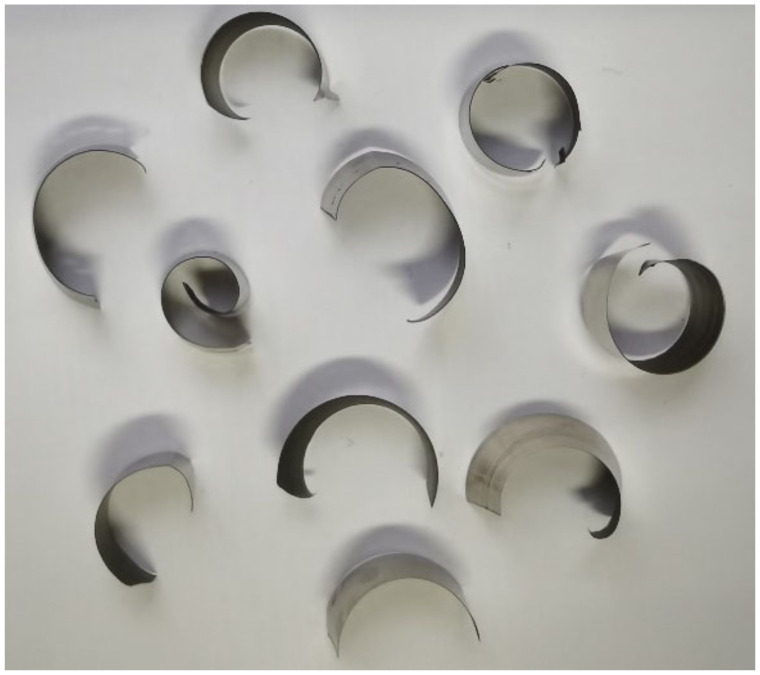
Chips.

The disc milling cutter cut the workpiece (Shaanxi Changyu Aviation Equipment Co., Ltd., Xi’an, China) without cutting fluid in Figure 5 in a symmetrical way, which means the workpiece was clamped in the symmetrical direction of the axial trajectory of the milling cutter. The chip is shown in Figure 6. It can be seen that the cutting type was a C-shaped chip.

## 4. Qualitative Relationship between Chip Parameters and Cutting Parameters

### 4.1. Chip Radius

**Figure 7:**
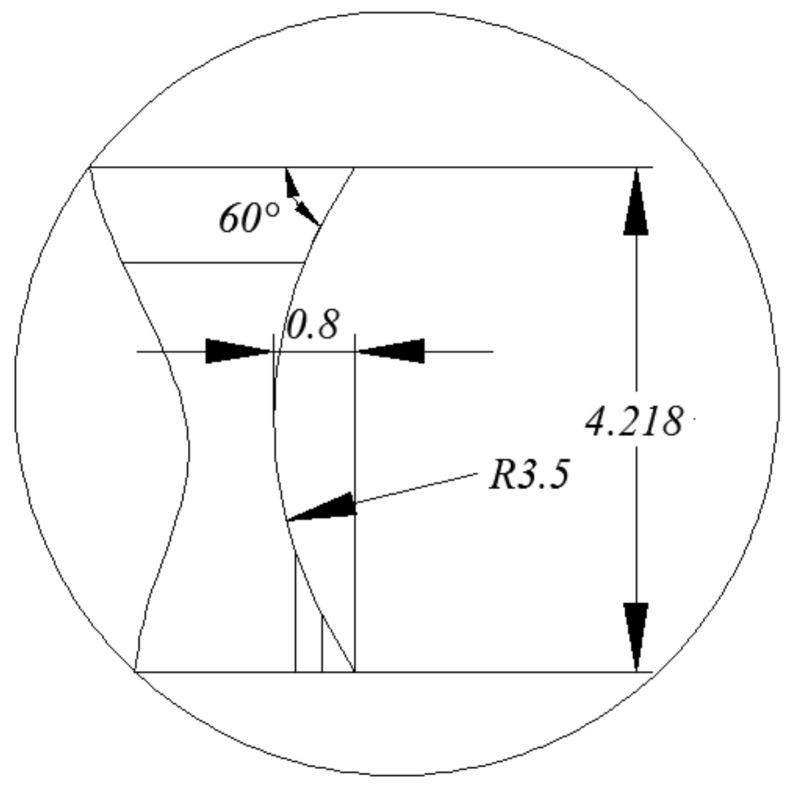
Geometric dimension of the chip breaking groove.

**Figure 8:**
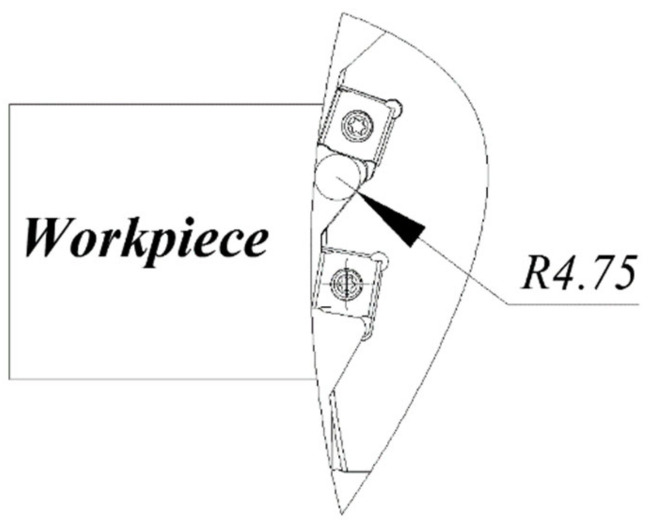
Geometric dimension of the chip pocket for the disc cutter.

**Figure 9:**
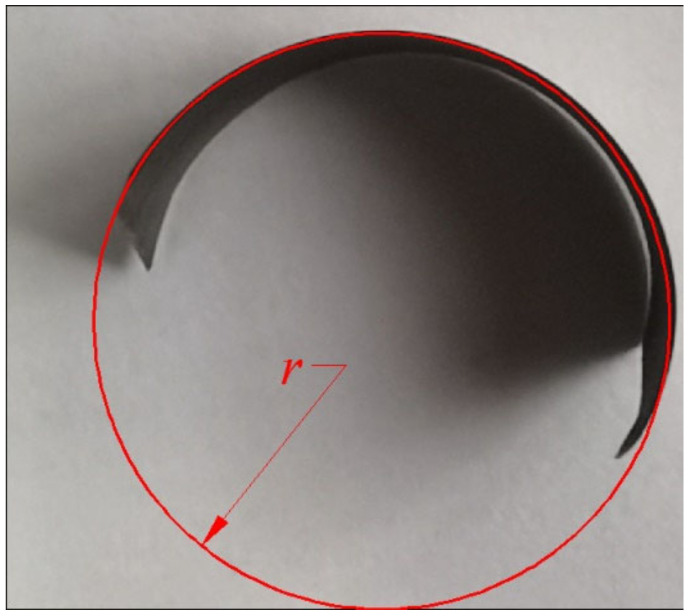
Measurement of chip curl radius.

The geometric dimension of the chip breaking groove of the inserts is shown in Figure 7, and the geometric dimension of the chip pocket for the disc cutter is shown in Figure 8. The radius of the chip breaking groove was 3.5 mm, and the radius of the chip pocket was 4.75 mm. So, the cutting radius was about 4.75 mm. Measurement of chip curl radius is shown in Figure 9. The measured chip radii are listed in Table 4.

### 4.2. Chip Thickness Deformation Coefficient

The chip thickness deformation coefficient *k_t_* is defined as the ratio of chip thickness *h_c_* to theoretical instantaneous cutting thickness *h_t_*, which can be expressed as

(1) $$k_{t}=\frac{h_{c}}{h_{t}}$$

The chip thickness deformation coefficients obtained in experiments are listed in Table 4.

### 4.3. Chip Width Deformation Coefficient

The chip width deformation coefficient *k_w_* is defined as the ratio of chip width *w_c_* to cutting depth *a_p_*, which can be expressed as

(2) $$k_{w}=\frac{w_{c}}{a_{p}}$$

The chip width deformation coefficients obtained in experiments are listed in Table 4.

### 4.4. Study on the Relationship between Chip Parameters and Cutting Parameters

**Figure 10:**
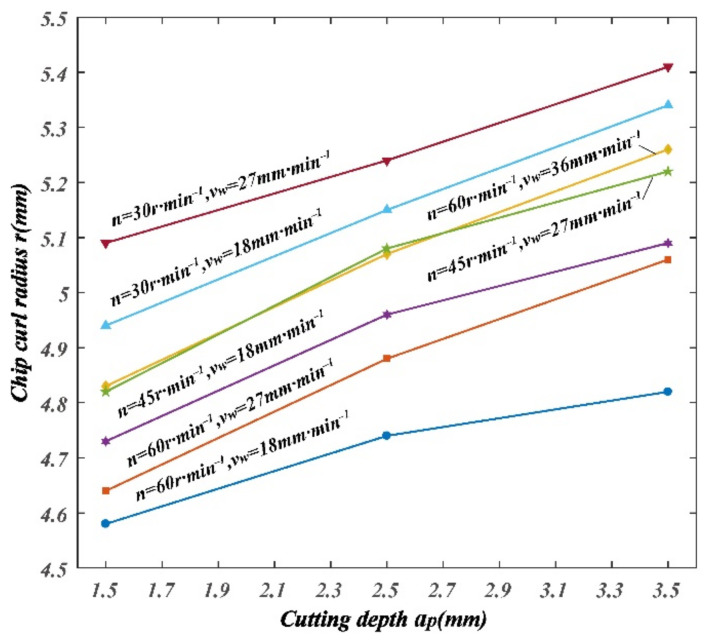
Variation of chip curl radius with cutting parameters.

**Figure 11:**
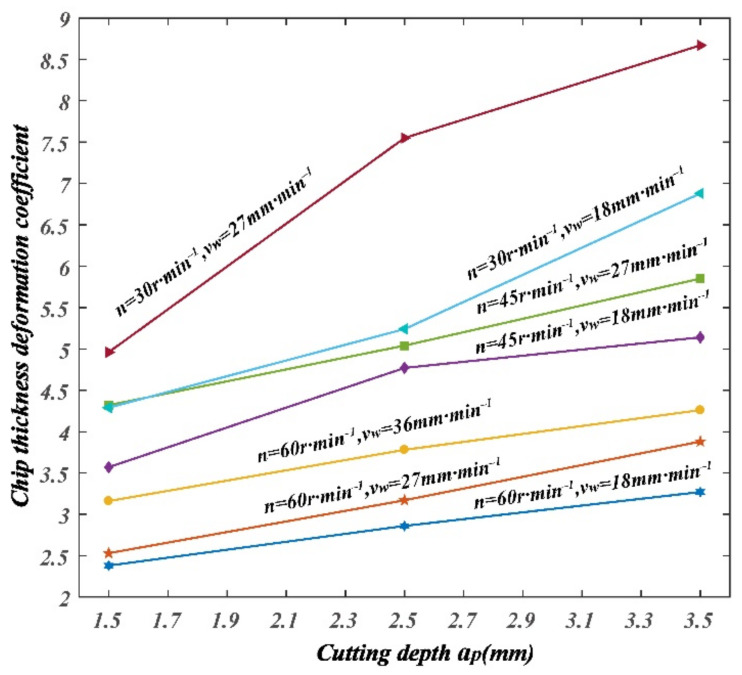
Variation of chip thickness deformation coefficient with cutting parameters.

**Figure 12:**
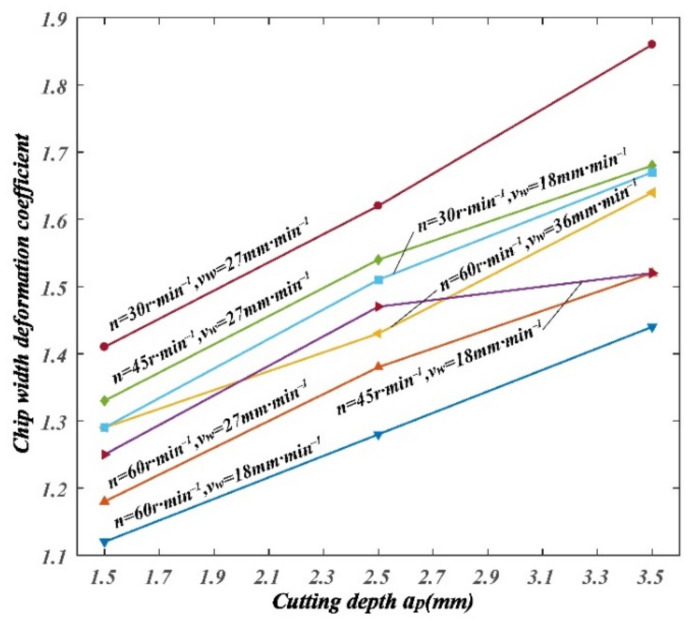
Variation of chip width deformation coefficient with cutting parameters.

The variation of chip curl radius, chip thickness deformation coefficient, and chip width deformation coefficient with cutting parameters can be drawn from Table 4, as shown in Figure 10, Figure 11 and Figure 12.

It can be seen from Figure 10 that the chip curl radius decrease was related to the increase in spindle speed; chip curl radius increase was related to the increase in feed speed; chip curl radius increase was related to the increase in cutting depth.

It can be seen from Figure 11 that the chip thickness deformation coefficient decreased with the increase in spindle speed; chip thickness deformation coefficient increased with the increase in feed speed; chip thickness deformation coefficient increased with the increase in cutting depth.

It can be seen from Figure 12 that the chip width deformation coefficient decreased with the increase in spindle speed; chip width deformation coefficient increased with the increase in feed speed; chip thickness width coefficient increased with the increase in cutting depth.

## 5. Mathematical Model for Chip Parameters

The quadratic polynomial has high fitting accuracy and good prediction accuracy. According to the experimental results in Table 4, the quadratic polynomial model of chip curl radius, chip thickness deformation coefficient, and chip width deformation coefficient can be expressed as

(3) $$\begin{array}{l} r=5.185-0.0191n-0.0157v_{w}+0.2792a_{p}+\\ 0.000033n^{2}+0.000324v_{w}^{2}-0.0293a_{p}^{2}+\\ 0.00016nv_{w}-0.000448na_{p}+0.00294v_{w}a_{p} \end{array}$$

(4) $$\begin{array}{l} k_{t}=-3.28+0.1399n+0.15v_{w}+2.758a_{p}-\\ 0.00063n^{2}+0.00236v_{w}^{2}-0.119a_{p}^{2}-\\ 0.00457nv_{w}-0.03553na_{p}+0.0172v_{w}a_{p} \end{array}$$

(5) $$\begin{array}{l} k_{w}=0.572+0.01086n+0.0116v_{w}+\\ 0.285a_{p}-0.000111n^{2}+0.000103v_{w}^{2}-0.0186a_{p}^{2}-\\ 0.000222nv_{w}-0.001543na_{p}+0.00231v_{w}a_{p} \end{array}$$

**Figure 13:**
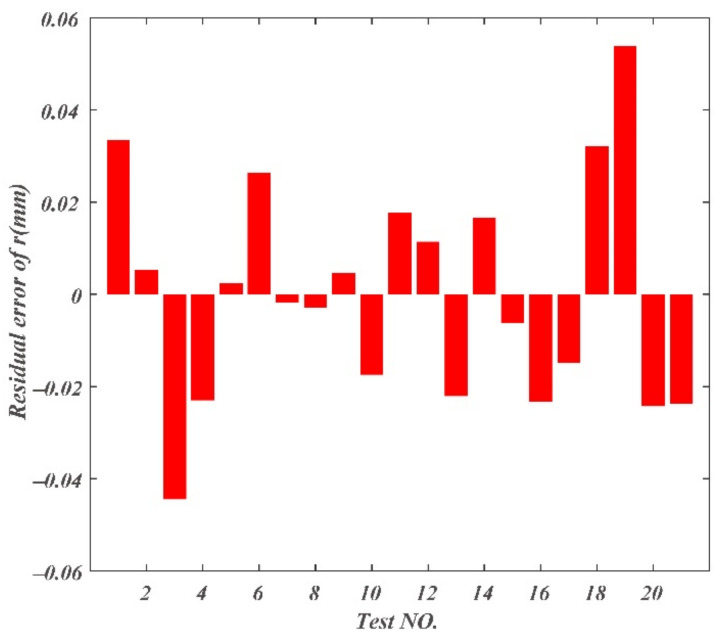
Residual errors of chip curl radius.

**Figure 14:**
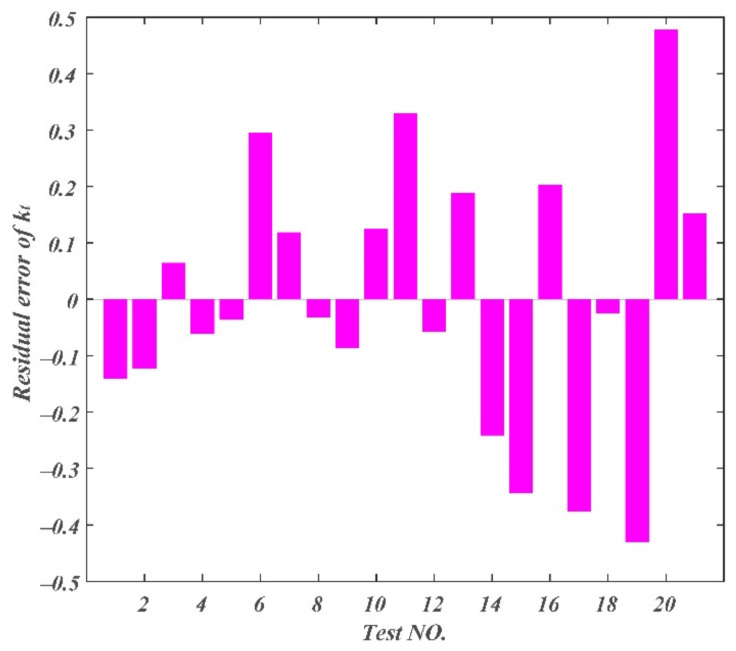
Residual errors of chip thickness deformation coefficient.

**Figure 15:**
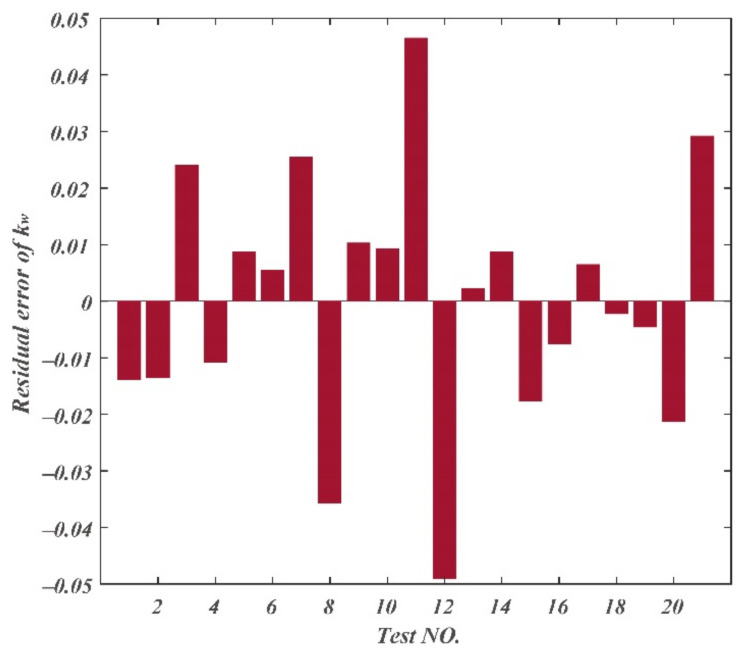
Residual errors of chip width deformation coefficient.

The residual errors of chip curl radius, chip thickness deformation coefficient, and chip width deformation coefficient are displayed in Figure 13, Figure 14 and Figure 15. The maximum residual error value of chip curl radius was less than 0.054 mm; the maximum residual error value of chip thickness deformation coefficient was less than 0.5; the maximum residual error value of chip width deformation coefficient was less than 0.05.

## 6. Experimental Verification

**Figure 16:**
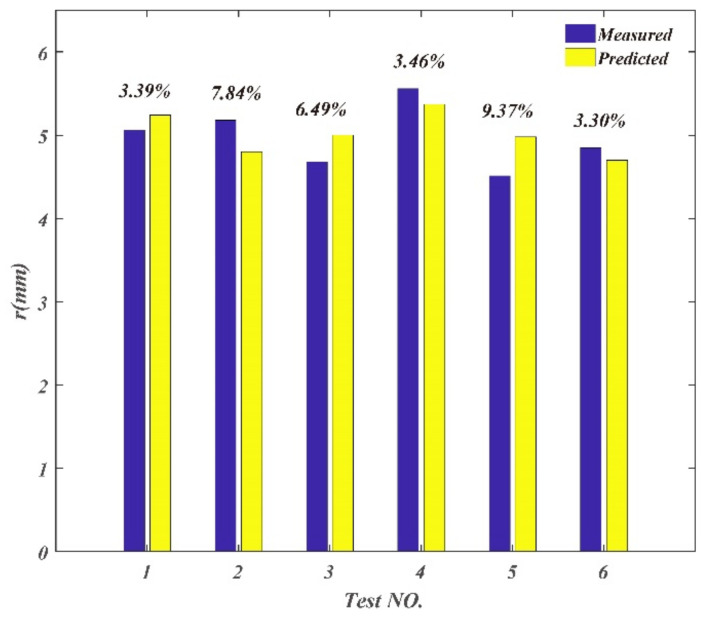
Comparison between the measured and predicted values for chip thickness deformation coefficient.

**Figure 17:**
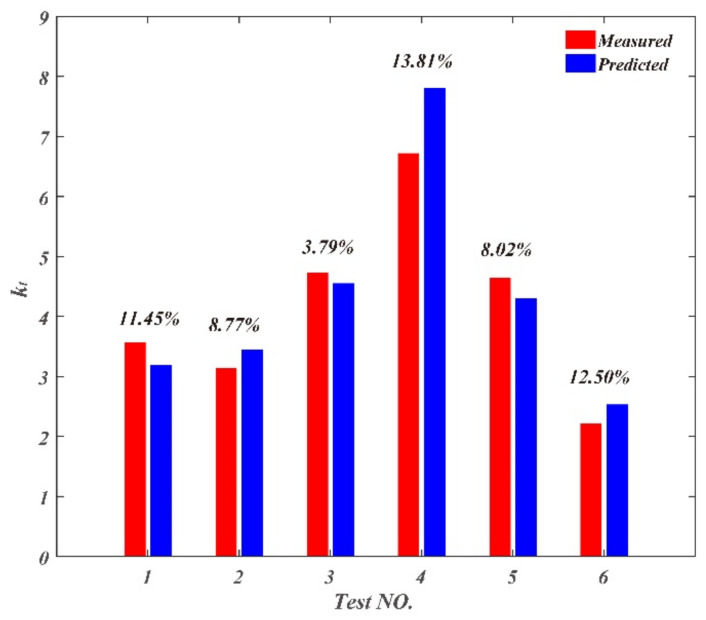
Comparison between the measured and predicted values for chip curl radius.

**Figure 18:**
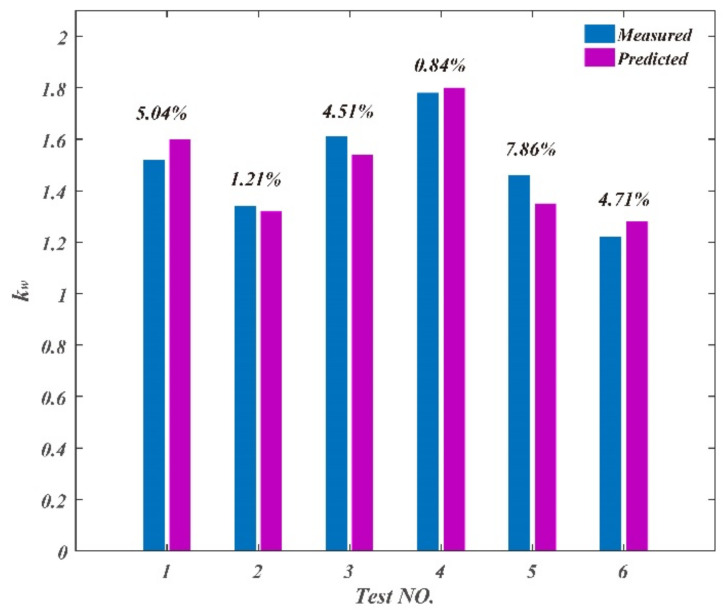
Comparison between the measured and predicted values for chip width deformation coefficient.

The validation parameters and results of chip parameters are shown in Table 5. The comparison between the measured and predicted values is shown in Figure 16, Figure 17 and Figure 18. The maximum deviation rate between measured values and predicted values for chip curl radius was 9.37% and the average deviation rate was 5.64%; the maximum deviation rate for cutting thickness deformation coefficient was 13.8% and the average deviation rate was 9.72%; the maximum deviation rate of cutting width deformation coefficient was 7.86% and the average deviation rate was 4.03%. The results show that the average deviation rates between measured values and predicted values for chip parameters were less than 10%, which indicates measured values are in good agreement with the predicted values. The average deviation rate for cutting thickness deformation coefficient was the largest of the three chip parameters, which means that there are more factors affecting cutting thickness deformation besides cutting parameters.

## 7. Discussion and Conclusions

Compared with the previous literatures, this paper studied the chip parameters in milling GH4169 with a special tool in a special machine tool. In this paper, the qualitative relationships between chip parameters for cutting GH4169 with an indexable disc milling cutter and cutting parameters were studied, and the quadratic polynomial models between chip parameters and cutting parameters were established. The results showed that the chip parameters (chip curl radius, chip thickness deformation coefficient, and chip width deformation coefficient) were negatively correlated with spindle speed; chip parameters were positively correlated with feed speed; chip parameters were positively correlated with cutting depth. The experimental verification indicated maximum deviation rate between measured values and predicted values for chip curl radius was 9.37%; the maximum deviation rate for cutting thickness deformation coefficient was 13.8%, and the maximum deviation rate of cutting width deformation coefficient was 7.86%. On the one hand, the model did not consider the insert eccentricity, deformation, and wear, as well as various assembly and manufacturing errors. On the other hand, the model established is only suitable for the processing combination of a specific machine tool, specific tool, and specific material, and the applicability of the model is poor. In the future, the model needs to be modified on the basis of considering insert wear and eccentricity high.

## Figures and Tables

**Table 1:** Main chemical constituents of GH4169 (%).

Ni	Cr	Mo	Nb	Ti	Al	C	Si	Mn	Fe
51.75	17	2.93	5.15	1.07	0.45	0.042	0.21	0.03	21.368

**Table 2:** Physical and mechanical properties of GH4169.

Physical Properties	Density (g/cm^3^)	Poisson’s Ratio	Thermal Conductivity (W/mK)	Specific Heat Capacity (J/kg °C)	Elastic Modulus (GPa)
	8.24	0.3	14.7	435	199.9
**Mechanical** **Properties**	**Elongation** **(%)**	**Reduction of Area** **(%)**	**Tensile Strength** **(MPa)**	**Impact Toughness** **(MJ/m^2^)**	**Yield Stress** **(MPa)**
	24	40	1430	348	1100

**Table 3:** Cutters and insert geometric parameters.

Number of Teeth	Diameter (mm)	Thickness (mm)	Rake Angle (°)	Flute Length (mm)	Angle of Inclination (°)
39	420	15	8	6	±2

**Table 4:** Results of chip parameters.

No.	*n*	*v_w_*	*a_p_*	*r*	*k_t_*	*k_w_*
1	60	18	1.5	4.58	2.38	1.12
2	60	18	2.5	4.74	2.86	1.28
3	60	18	3.5	4.82	3.27	1.44
4	60	27	1.5	4.64	2.53	1.18
5	60	27	2.5	4.88	3.17	1.38
6	60	27	3.5	5.06	3.88	1.52
7	60	36	1.5	4.83	3.16	1.29
8	60	36	2.5	5.07	3.78	1.43
9	60	36	3.5	5.26	4.26	1.64
10	45	18	1.5	4.73	3.57	1.25
11	45	18	2.5	4.96	4.77	1.47
12	45	18	3.5	5.09	5.14	1.52
13	45	27	1.5	4.82	4.32	1.33
14	45	27	2.5	5.08	5.04	1.54
15	45	27	3.5	5.22	5.85	1.68
16	30	18	1.5	4.94	4.29	1.29
17	30	18	2.5	5.15	5.24	1.51
18	30	18	3.5	5.34	6.88	1.67
19	30	27	1.5	5.09	4.96	1.41
20	30	27	2.5	5.24	7.55	1.62
21	30	27	3.5	5.41	8.67	1.86

**Table 5:** Validation parameters and results of chip parameters.

No.	*n*	*v_w_*	*a_p_*	*r*	*k_t_*	*k_w_*
1	65	35	4	5.06	3.56	1.52
2	55	25	2	5.18	3.14	1.34
3	50	15	4	4.68	4.72	1.61
4	40	35	3	5.56	6.72	1.78
5	35	15	2	4.51	4.64	1.46
6	65	15	3	4.85	2.21	1.22

## Data Availability

No new data were created or analyzed in this study. Data sharing is not applicable to this article.

## Nomenclature

*n*	Spindle speed, r/min
*v_w_*	Feed speed, mm/min
*a_p_*	Cutting depth, mm
*r*	Chip curl radius, mm
*k_t_*	Chip thickness deformation coefficient
*k_w_*	Chip width deformation coefficient
*h_c_*	Chip thickness, mm
*h_t_*	Theoretical instantaneous cutting thickness, mm
*w_c_*	Chip width, mm
